# Recent advances in the understanding of endometriosis: the role of inflammatory mediators in disease pathogenesis and treatment

**DOI:** 10.12688/f1000research.7504.1

**Published:** 2016-02-17

**Authors:** Warren Nothnick, Zahraa Alali

**Affiliations:** 1 Department of Molecular and Integrative Physiology, University of Kansas Medical Center, Kansas City, KS, USA; 2 Institute for Reproductive Health and Regenerative Medicine, Center for Reproductive Sciences, University of Kansas Medical Center, Kansas City, KS, USA

**Keywords:** endometriosis, prostaglandin E2, estrogen receptor-beta

## Abstract

In this review, we focus on recent advancements in our understanding of the roles of inflammatory mediators in endometriosis pathophysiology and the potential for improved therapies based upon targeting these pathways. We review the association between endometriosis and inflammation and the initial promise of anti-tumor necrosis factor therapies based upon experimental evidence, and how and why these studies have not translated to the clinic. We then discuss emerging data on the role of inter-relationship among macrophage migration inhibitory factor, prostaglandin E _2_, and estrogen receptor-beta, and the potential utility of targeting these factors in endometriosis treatment. In doing so, we highlight the strengths and discuss the current research on identification of novel, anti-inflammatory-based therapy and the necessity to expand experimental endpoints to include clinically relevant measures when assessing the efficacy of potential new therapies for endometriosis.

## Introduction

In this short report, we focus on recent advances in our understanding on the role of inflammatory mediators in the pathophysiology of endometriosis and the potential utility of therapeutic agents that target their action. We discuss how initial studies of related targets, such as tumor necrosis factor-alpha (TNF-α), failed to result in novel, non-hormonal therapy, and we introduce new players which have gained attention for their role in the pathophysiology of endometriosis. We discuss emerging data on the role and inter-relationship among macrophage migration inhibitory factor (MIF), prostaglandin E _2_ (PGE _2_), and estrogen receptors alpha (ER-α) and beta (ER-β), and the potential utility of targeting these factors in endometriosis treatment. We highlight the strengths of current research on the identification of novel, anti-inflammatory-based therapy and discuss the necessity to expand experimental endpoints to include clinically relevant measures when assessing the efficacy of potential new therapies for endometriosis.

## Endometriosis and limitations with current therapies

Endometriosis is a disease which affects women of reproductive age and is defined as the growth of endometrial tissue in ectopic locations, primarily within the pelvic cavity. Endometriosis is a chronic disease characterized by pelvic pain and infertility, and affects over 70 million women worldwide. Given a 10% prevalence rate among women of reproductive age, the annual costs of endometriosis were estimated at $22 billion in 2002 in the US alone. These costs are considerably higher than those related to migraine or Crohn’s disease ^1^. One reason for the high cost is that there are insufficient treatments for the disease. Current medical treatment approaches rely on the fact that endometriosis is an estrogen-dependent disease. Yet, relief is at the expense of induction of a hypo-estrogenic state, which is counterproductive for infertility treatment and associated with unwanted menopausal-like side effects, the major drawback being a potential reduction in bone density.

These drawbacks of current endometriosis treatments often lead to abandonment of medical therapy and repeated surgical therapy. As such, there is a great need for the identification of novel targets for endometriosis treatment. Treatments that directly target the endometriotic implant would overcome the abovementioned shortcomings. Unfortunately, there are no current endometriotic implant-specific treatment options that have been shown to be successful or act independently of steroid production or action. The potential of targeting inflammatory mediators associated with endometriosis has been intensely investigated over the past three decades with varying degrees of success. The purpose of the following review is to provide a brief history of how these targets emerged as potential, non-hormonal-based therapies, outline what we learned from prior studies, and discuss why early therapeutics have yet to show efficacy. From there, we highlight the study of emerging mediators of inflammation associated with endometriosis and define the cooperative interaction among these mediators in the pathophysiology of endometriosis, as well as discuss the future application of targeting these mediators toward development of novel, anti-inflammatory therapeutic agents for endometriosis treatment.

## Endometriosis, inflammation, and progesterone resistance

It has long been acknowledged by both researchers and clinicians that endometriosis is a disease associated with inflammation and elevated cytokine levels ^2, 3^. Altered cytokine production by both cells of the immune system and the endometriotic lesion tissue has been proposed (discussed below for each of the specified cytokines) to contribute to these elevated cytokine levels. One of the driving factors for the enhanced production of endometriotic lesion cytokines is an altered progesterone responsiveness associated with the disease. Progesterone exhibits anti-inflammatory actions, and as such, progesterone analogs have been used to treat endometriosis and its associated symptoms ^4^. Progestin (progesterone) treatment appears to be successful in most ^5^, but not all ^6^, women, and not all progestin formulations are effective in reducing endometriosis-associated pain ^7^. This inconsistency could be due to the progesterone resistance typical of endometriosis which may stem from altered progesterone receptor expression ^8^.

For example, expression of progesterone receptors PR-A and PR-B is altered in endometriotic lesion stromal cells. More specifically, compared with eutopic endometrium, PR-A is markedly reduced and PR-B is absent ^9^. Not only does reduced expression of receptors for this steroid dampen the ability to suppress cytokine production, but reduced progesterone action contributes to elevated local estrogen levels which further drive the endometriotic lesion phenotype and elevated cytokine levels. With this in mind, there has been considerable investigation over the past 20 years examining the roles of specific immune/inflammatory mediators and the potential to target these molecules as novel, estrogen-sparing treatments for this disease. Unfortunately, despite this vast effort, there is still a general sense of uncertainty on which immune/inflammatory mediators appear to be key players in the pathophysiology of endometriosis and the efficacy of targeting these molecules as endometriosis treatment options.

## Tumor necrosis factor and endometriosis

TNF-α, a pro-inflammatory cytokine, was one of the early non-hormonal targets for potential endometriosis therapy ^10– 13^. It was first demonstrated to be elevated in the peritoneal fluid ^14– 17^ and serum ^16, 17^ of women with endometriosis but is now known to be produced by several cell types ^18^, including cells of the endometriotic lesions ^19^. *In vitro* studies demonstrated that this cytokine stimulated cellular events conducive to the establishment and progression of endometriosis, such as adhesion and induction of protease and inflammatory mediators ^20– 22^.

Based upon these observations, initial studies evaluated the efficacy of targeting TNF-α as a potential treatment for endometriosis. The first studies tested a recombinant human TNF-α-binding protein (rhTBP-1) ^10^ in a rat model of endometriosis; these were followed by a series of studies using rhTBP-1 in a baboon model of endometriosis ^11– 13^. Unfortunately, studies showing a reduction in disease burden in experimental models have not paralleled studies on efficacy of anti-TNF-α therapy for endometriosis symptomology, as summarized by Lu and colleagues in a recent Cochrane Database review ^23^. Thus, studies on the use of anti-TNF-α have stalled and no new data have emerged to support the use of such compounds for the treatment of symptomatic endometriosis.

The discrepancy between the encouraging results reported in experimental animal model studies and the lack of an effect detected in clinical trials likely stems from the differences in endpoint analysis. Experimental animal model studies focused primarily on reduced disease burden/lesion size, whereas the clinical trials have focused on the alleviation of pain. Unfortunately, it is unclear whether anti-TNF-α therapy reduced disease burden (stage of endometriosis) in women who received these compounds. We do know from these trials that anti-TNF-α therapy does not reduce pain, which is a chief complaint associated with the disease. The fact that pain is a symptom that is strongly associated with disease presence, but not with disease burden, does not allow conclusions to be drawn with respect to potential impact (or lack of impact) on disease stage in these patients. In animal models, although we do know there is a reduction in disease burden, we do not know whether there is a reduction in pain in those animals treated with anti-TNF-α therapies. Induction of experimental endometriosis in animal models has been demonstrated to elicit pain, initially described in rats by independent groups ^24, 25^ and more recently in a mouse model ^26^. Unfortunately, these early studies on anti-TNF-α therapy were conducted prior to the validation of rodent models of pain assessment in animals with experimentally induced endometriosis. One lesson from these studies is that a focus on multiple clinically relevant endpoints in the animal models would be of benefit. Another lesson is that we lack non-surgical clinical biomarkers of disease burden that would be of great use in human studies.

Despite this uncertainty on the role and potential therapeutic benefits of targeting inflammatory mediators such as TNF-α, there is still considerable interest in studying the role of pro-inflammatory mediators in the pathogenesis of endometriosis and the potential benefit of targeting these molecules. Although the initial excitement of anti-TNF-α therapy has waned, additional research on other mediators of inflammation has intensified. Targets getting increased attention are MIF and PGE2.

## Macrophage migration inhibitory factor and endometriosis

Like TNF-α, MIF is elevated in the peritoneal fluid ^27^, circulation ^28^, and peritoneal macrophages from women with endometriosis ^29^. MIF is also expressed in active and early/stage I endometriotic lesions ^30^, as well as overexpressed in eutopic endometrium in women with the disease ^31^. Within endometriotic lesion cells, MIF is induced by estrogen ^32^, and we have recently demonstrated that MIF expression is associated with endometriotic lesion survival status in women with the disease ^33^.

MIF was originally identified as a potent mitogenic factor for human endothelial cells *in vitro* and tumor angiogenesis *in vivo* ^34^. Yang and colleagues demonstrated that, in patients with endometriosis, MIF could stimulate endothelial cell proliferation ^35^. Further supporting a role of MIF in endometriotic lesion survival, MIF has been shown to stimulate PGE2, COX-2 ^36^, vascular endothelial growth factor (VEGF), interleukin-8 (IL-8), and monocyte chemotactic protein-1 (MCP-1) expression ^37^, as well as the induction of aromatase expression in a feed-forward mechanism ^32^. Interestingly, MIF also stimulates TNF-α secretion ^38^, whereas TNF-α is also capable of inducing MIF production ^39^ in endometrial cells. Thus, it is tempting to speculate that a feed-forward amplification of these cytokines and their downstream pathways exists in endometriosis. Also of relevance to the pathophysiology of endometriosis is the demonstration that many of these MIF-induced factors are associated with a proliferative and angiogenic phenotype conducive to endometriotic establishment or growth (or both) ^37^. As such, there is ample evidence to suggest a strong association between elevated MIF expression/levels and endometriosis *in vivo,* as well as *in vitro* evidence which indicates that MIF can induce factors which are believed to be essential for endometriosis development and survival.

Building upon these initial observations, several studies have evaluated the efficacy of targeting MIF as a potential endometriosis treatment. In 2011, we first reported the utility of targeting MIF as a potential therapy for endometriosis ^40^. In that study, we used an experimental mouse model of endometriosis in which the females were immune-competent and reproductively intact (non-ovariectomized and non-estrogen-supplemented) and harbored endometriotic lesions derived from donor wild-type mice. We demonstrated that the MIF antagonist, ISO-1, could induce a significant reduction in lesion size. Of potential clinical significance was the finding that ISO-1 reduced lesion burden without affecting reproductive cyclicity or presumed estrogen action ^40^. Using a mouse model for endometriosis in which immune-compromised mice harbored endometriotic lesions derived from human tissue, Khoufache and colleagues ^41^ demonstrated a similar ability of ISO-1 to decrease the number, size, and dissemination of endometriotic lesions. Furthermore, they demonstrated that inhibition of MIF by ISO-1 impedes lesion dynamics by inhibiting cell adhesion, tissue remodeling, angiogenesis, and inflammation, in addition to altering the balance of pro- and anti-apoptotic factors ^41^. More recently, this group provided additional proof of principal by using an ovariectomized, estrogen-supplemented mouse model for endometriosis incorporating *Mif*-deficient mice as both tissue recipient and tissue donors ^42^. Consistent with previous studies in mouse models ^40, 41^, both pharmacologic inhibition of MIF (with ISO-1) and genetic ablation of *Mif* ( *Mif*-deficient mice) induced a reduction in lesion burden. Of notable interest was the demonstration that *Mif*-deficient hosts that harbored either normal (expressing *Mif*) or *Mif*-deficient lesions had impaired lesion growth, strongly suggesting the critical importance of *Mif* in the pathogenesis of endometriosis.

Initial studies evaluating ISO-1 as a therapeutic agent for endometriosis treatment are encouraging as the MIF antagonist reduces lesion burden in mouse models which harbor both mouse and human tissue, demonstrating efficacy. Furthermore, this inhibitory effect of MIF antagonism occurs independently of reproductive cyclicity/estrogen levels and action, and may permit continuation of reproductive cycles while relieving disease burden. Clearly, studies are warranted to evaluate whether these beneficial effects of ISO-1 can be extended to alleviating the pain associated with endometriosis in animal models with the extension of MIF antagonist into clinical trials.

## Prostaglandin E _2_ and endometriosis

In addition to regulating cytokine production, MIF has been shown to stimulate PGE _2_ production ^43^. PGE _2_ has been proposed as a master regulator of endometriosis ^44^ on the basis of its pro-inflammatory actions. PGE _2_ and the biosynthesis enzymes responsible for its liberation are elevated in human endometriotic lesion tissue ^45, 46^ as well as peritoneal macrophages ^47^ and peritoneal fluid ^48^ from women with endometriosis. *In vitro* studies support a role for PGE _2_ in the mechanisms conducive to endometriosis establishment and survival. For example, selective inhibition of the PGE _2_ receptors, prostanoid receptor-2 and (EP2) and EP4, inhibits cellular adhesion, invasion, growth, and survival of human endometriotic epithelial and stromal cells *in vitro* ^49– 52^.

Inhibition of PGE _2_ action has also been associated with favorable outcome in experimental animal models of endometriosis ^26, 53^. Using a hamster model of endometriosis, Laschke and colleagues ^53^ demonstrated that administration of the selective COX-2 inhibitor, NS398, induced a marked regression of ectopic lesions by inhibiting angiogenesis and suppressing cellular proliferation and inducing apoptosis. More recently, Arosh and colleagues ^26^ incorporated mouse models of endometriosis and demonstrated that selective inhibition of the PGE _2_ receptors EP2/EP4 decreased growth and survival, as well as angiogenesis and innervation of ectopic lesions. Furthermore, inhibition of PGE _2_ signaling was associated with suppression of the pro-inflammatory state of dorsal root ganglia neurons and decreased pelvic pain as well as a decrease in the pro-inflammatory, estrogen-dominant, and progesterone-resistant molecular environment of the eutopic endometrium and ectopic lesions. There are also clinical data which demonstrate that use of rofecoxib, a COX-2 inhibitor (at 25 mg per day for 6 months), resulted in a significant improvement in pelvic pain and dyspareunia after the course of treatment in women with disease, both by comparison with pre- and post-treatment as well as compared with pain assessment in subjects receiving placebo only ^54^. Given that PGE2 is induced by both MIF and TNF-α, it is tempting to speculate that inhibition of these cytokines and the reduction in lesion burden may have been due at least in part to reduction in PGE2 levels or action (or both).

## Estrogen receptor-beta and endometriosis

As mentioned earlier in this review, it is well established that endometriosis is an estrogen-dependent disease and that there is a strong connection between estrogen and the inflammatory environment associated with the disease. However, the complex downstream mediators which impart the pathophysiology of the disease are only partially understood. ER-β is one of the two nuclear receptors that mediate estrogen action. Within the context of endometriosis, ER-β is significantly higher (over 100-fold) in endometriotic lesion tissue compared with eutopic endometrium ^55– 57^, and this may be due to altered methylation in the gene promoter ^57^. This overexpression of ER-β leads to a decrease in ER-α expression ^58^, resulting in an abnormally high ER-β-to-ER-α ratio which is associated with elevated endometriotic lesion COX-2 levels ^59^. Activation of ER-β has also been demonstrated to induce MIF ^32^ expression by endometriotic lesion cells. Thus, estrogen acting through ER-β-stimulated pathways may play a role in the pathophysiology of endometriosis. Given that endometriosis is an estrogen-dependent disease, inhibition of this pathway might be anticipated to suppress lesion survival and symptoms of endometriosis. If so, one would anticipate that inhibition of ER-β-mediated signaling, though effective in reducing endometriotic lesion burden, may also be associated with an induction of a hypo-estrogenic state, with resultant adverse effects, including menopausal signs and symptoms and loss of reproductive cyclicity.

Three studies to date have evaluated the use of ER-β ligands in the potential treatment of endometriosis using experimental animal models. An early study by Harris and colleagues ^60^ used an ER-β-specific agonist (ERB-041) in an experimental mouse model of endometriosis and reported a regression of ectopic lesion growth. Assessment of lesion tissue (derived from human endometrium) revealed a lack of ER-β expression, leading the authors to conclude that ERB-041 exerted its effects on the host immune system, rather than on the implanted tissue, possibly by induction of apoptosis. Unfortunately, the investigators did not elaborate on the mechanism by which this occurred. As activation of ER-β decreases ER-α expression ^58^, it may be possible that downregulation of ER-α contributed to these observations.

More recently, Zhao and colleagues ^61^ elegantly dissected the role of both ER-α and ER-β signaling by using an experimental mouse model of endometriosis incorporating the novel ER ligands chloroindazole (CLI) (exhibits ER-β-dependent activity) and oxabicycloheptene sulfonate (OBHS) (greater ER-α-preferential binding selectivity) which exhibit both anti-estrogenic and anti-inflammatory activity. Most importantly, both CLI and OBHS induced lesion regression and suppression of inflammatory events associated with endometriosis without disrupting normal reproductive cyclicity and fertility. Thus, the anti-estrogenic/antagonistic effect of these ligands suggests that the ER-β (and ER-α) pathway is involved in the pathogenesis of endometriosis, and that the effects of estrogen antagonism can be separated between those that impact inflammation and lesion regression and those that regulate reproductive cyclicity and fertility.

This postulate is further supported by the work of Han and colleagues ^62^, who demonstrated that activation of the ER-β pathway may contribute to the pathogenesis of endometriosis. Using experimental mouse models of endometriosis which incorporate genetically modified mice in which ER-α and ER-β are conditionally deleted, these investigators demonstrated that use of the ER-β antagonist, PHTPP, was associated with a regression of ectopic lesions. These investigators went on to dissect the mechanism by using experimental endometriosis models that incorporated genetically modified mice which either overexpressed ER-β, or had ER-α or ER-β (or both) deleted from uterine/endometriotic tissue. This study demonstrated that ER-β is responsible for inhibiting endometriotic cell apoptosis and increases cytokine production to enhance cellular adhesion and proliferation as well as enhance epithelial-mesenchymal transition signaling to increase cell invasion. As suggested by Han and colleagues, and supported by the study by Zhoa and colleagues ^61^, targeting ER-β may have beneficial effects on lesion growth/survival as well as the potential to improve infertility at the level of the eutopic endometrium ^62^, or at least spare reproductive competency while reducing lesion burden. The potential efficacy of targeting ER-β/ER-α with these novel ligands to reduce pain associated with endometriosis remains to be determined, as does the assessment of the potential impact on bone density.

## Summary

**Figure 1.**
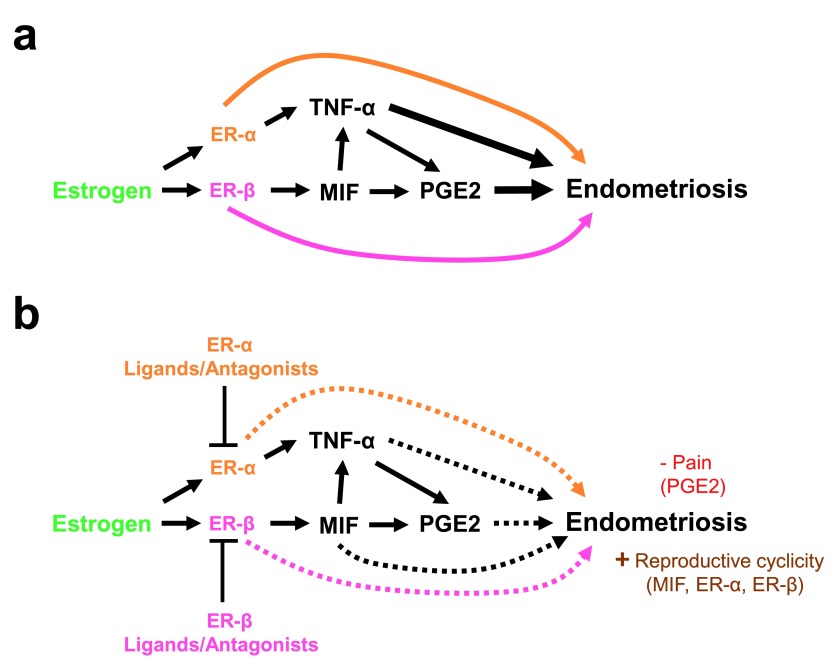
Estrogen regulation of inflammatory mediators in the pathophysiology of endometriosis. ( **a**) Estrogen stimulates the establishment, growth, and survival of endometriotic tissue through the induction of tumor necrosis factor-alpha (TNF-α), macrophage migration inhibitory factor (MIF), and prostaglandin E2 (PGE2) (black arrows) as well as through other estrogen receptor-alpha (ER-α)- and ER-β-dependent pathways (orange and pink arrows, respectively). ( **b**) Inhibition of estrogen, TNF-α, MIF, and PGE2 leads to reduced endometriosis burden in experimental animal models of endometriosis. Broken lines indicate inhibition of endometriotic lesion burden by antagonism of estrogen, TNF-α, MIF and/or PGE2 signaling. “- Pain” indicates those specified antagonists which were demonstrated to reduce lesion burden and pain in experimental animal models of endometriosis. “+ Reproductive cyclicity” indicates those specified antagonists which reduced endometriosis burden but did not negatively impact reproductive cyclicity/fertility in experimental animal models of endometriosis.

For over two decades, the role of inflammatory mediators and the potential to target them as a non-hormonal means of treating endometriosis have been explored. Early studies focusing on TNF-α appeared promising on the basis of effects on lesions in experimental animal models but failed to produce clinical results on pain symptoms. More recent focus has turned toward MIF and PGE2 as potential targets for endometriosis treatment. Much like earlier studies focusing on TNF-α, experimental model studies have yielded promising results on their ability not only to suppress lesion growth but also to reduce pelvic pain, both independently of reproductive cyclicity. As yet, confirmatory studies in human subjects remain to be initiated. In addition to MIF and PGE2, the ER-β pathway has emerged as a potential target for endometriosis treatment. Of interest is the finding that the ER-β pathway appears to mediate many of the cytokines described in this review in modulating endometriotic lesion growth in animal models (summarized in Figure 1).

What we have learned in recent years is that estrogen action within the pathogenesis of endometriosis can be partitioned into inflammatory pathways that drive lesion survival and those steroid hormone pathways that modulate reproductive cyclicity. With recent advances in our ability to dissect the estrogen-regulated pathways by using novel pharmacologic and genetic tools, we have learned that the most effective estrogen-sparing target for endometriosis treatment may be an estrogen receptor itself.

## Abbreviations

CLI, chloroindazole; COX-2, cyclooxygenase-2; EP2, prostanoid receptor-2; EP4, prostanoid receptor-4; ER-α, estrogen receptor-alpha; ER-β, estrogen receptor-beta; ISO-1, (S,R)-3-(4-hydroxyphenyl)-4,5-dihydro-5-isoxazole acetic acid methyl ester; MIF, macrophage migration inhibitory factor (human); Mif, macrophage migration inhibitory factor (murine); OBHS, oxabicycloheptene sulfonate; PGE _2_, prostaglandin E _2_; PHTPP, 4-[2-Phenyl-5,7-bis(trifluoromethyl)pyrazolo[1,5-a]pyrimidin-3-yl]phenol; rhTMP-1, recombinant human tumor necrosis factor-alpha-binding protein; TNF-α, tumor necrosis factor-alpha.
